# Deoxynivalenol Biomarkers in the Urine of UK Vegetarians

**DOI:** 10.3390/toxins9070196

**Published:** 2017-06-22

**Authors:** Liz Wells, Laura Hardie, Courtney Williams, Kay White, Yunru Liu, Barbara De Santis, Francesca Debegnach, Georgio Moretti, Stephanie Greetham, Carlo Brera, Maria Papageorgiou, Natalie J. Thatcher, Alan Rigby, Stephen L. Atkin, Thozhukat Sathyapalan

**Affiliations:** 1 Department of Academic Diabetes, Endocrinology and Metabolism, Brocklehurst Building, Hull Royal Infirmary, Anlaby Road, Hull HU3 2RW, UK; liz.wells@hyms.ac.uk (L.W.); mar_papag@hotmail.com (M.P.); 2 Division of Epidemiology and Biostatistics, LICAMM, School of Medicine, University of Leeds, Leeds LS2 9JT, UK; l.j.hardie@leeds.ac.uk (L.H.); medcwila@leeds.ac.uk (C.W.); k.l.m.white@leeds.ac.uk (K.W.); 3 Department of Environmental Medicine, Hainan Medical University, 3 Xueyuan Road, Haikou 571199, China; liuyunru@126.com; 4 Department of Veterinary Public Health and Food Safety, GMO and Mycotoxins Unit, Istituto Superiore di Sanità, Viale Regina Elena, 299,001,61 Rome, Italy; barbara.desantis@iss.it (B.D.S.); francesca.debegnach@iss.it (F.D.); carlo.brera@iss.it (C.B.); 5 Department of Statistical Sciences, Piazzale Aldo Moro, Università degli studi di Roma “La Sapienza”, 500,185 Roma, Italy; giorgio.moretti87@gmail.com; 6 Echuca Regional Health, Service Street, Echuca 3564, Australia; sgreetham@erh.org.au; 7 European Food Safety Authority (EFSA), 43126 Parma, Italy; natalie.thatcher@gmail.com; 8 Centre for Cardiovascular and Metabolic Research, Hull York Medical School, Hertford Building, University of Hull, Hull HU6 7RX, UK; asr1960@hotmail.com; 9 Weill Cornell Medicine in Qatar, Education City, P.O. Box 24144, Qatar; sla2002@qatar-med.cornell.edu

**Keywords:** mycotoxins, deoxynivalenol, vegetarians, vomitoxin

## Abstract

Deoxynivalenol (DON) is produced by *Fusarium graminearum* and is one of the most commonly occurring trichothecenes. Vegetarians are alleged to be a high-risk group for DON exposure due to high intakes of cereals susceptible to the growth of the mycotoxin. This study provides the levels of DON and de-epoxi Deoxynivalenol (DOM-1) in urine analysed by liquid chromatography-mass spectrometry (LC-MS) in UK vegetarians. Over two consecutive days, morning urine samples were collected from 32 vegetarians and 31 UK adult volunteers, and associated food consumption 24 h prior to the sample was recorded. Statistically significant differences between the weight of the UK adults and vegetarians (*t* = 3.15. *df* = 61, *p* ≤ 0.005 two-tailed) were observed. The mean levels of DON in urine for adults on day 1 was 3.05 ng free DON/mg creatinine, and on day 2 was 2.98 ng free DON/mg creatinine. Even though high mean levels were observed, most adults were within the tolerable daily intake. However, for vegetarians, the mean level of urinary DON on day 1 was 6.69 ng free DON/mg creatinine, and on day 2 was 3.42 ng free DON/mg creatinine. These levels equate to up to 32% of vegetarians exceeding recommended tolerable daily intakes (TDI) of exposure (1 µg/kg b.w./day).

## 1. Introduction

Mycotoxins are produced by moulds of the genera *Penicillium, Fusarium,* and *Aspergillu*s which naturally occur in grains, causing cereal-based foodstuff contamination. The mycotoxin deoxynivalenol (DON) belongs to trichothecenes, which represents the main group of *Fusarium* toxins commonly found in cereal grains. The major contributors to DON exposure are cereal-based foodstuffs. Therefore, the evaluation of deoxynivalenol and its metabolites in urine may reflect an accurate measure of the dietary exposure. A temporary tolerable daily intake (TDI) of 1 μg/kg body weight (b.w.) was established by the EU Scientific Committee on Food [1] that was in accord with the temporary tolerable daily intake established by the Nordic Group [2] and the World Health Organisation [3]. In 2010, the Joint Expert Committee for Food Additives (JECFA) [4] stated that the provisional maximum tolerable daily intake for DON could be converted to a group PMTDI of 1 μg/kg b.w. for DON and its acetylated derivatives, 3-acetyl-deoxynivalenol (3-Ac-DON) and 15-acetyl-deoxynivalenol (15-Ac-DON) [4]. JECFA derived an acute reference dose (ARfD) of 8 μg/kg b.w of DON as a probable factor for acute pathologies in humans. In 2017, at the European Food Safety Authority’s 82nd plenary meeting on the contaminants in the food chain, the CONTAM panel adopted a draft opinion, reiterating the statement put forth by JECFA in 2010 [4].

The CONTAM Panel considered DON, 3-Ac-DON, 15-Ac-DON, and DON-3-glucoside, as they are currently the most relevant forms of DON in Europe occurring in food and feed, mainly in cereal grains and cereal-based food.

For the acute effects (vomiting) in humans, the CONTAM panel established a group acute reference dose (ARfD) of 8 μg/kg b.w. per eating occasion for the sum of DON, 3-Ac-DON, 15-Ac-DON, and DON-3-glucoside and for chronic effects a group tolerable daily intake (TDI) of 1 μg/kg b.w. per day for the sum of DON, 3-Ac-DON, 15-Ac-DON, and DON-3-glucoside.

It was concluded by the CONTAM panel that the current acute exposure to the sum of DON, 3-Ac-DON, 15-Ac-DON, and DON-3-glucoside in food raises no health concern. However, there is a health concern associated with chronic dietary exposure to the sum of DON, 3-Ac-DON, 15-Ac-DON, and DON-3-glucoside, particularly for infants, toddlers, and other children in some European countries. In adolescent and adult age groups, the group TDI was exceeded only at high chronic exposures.

DON and its metabolites may be measured in human urine following consumption of contaminated products as part of biomonitoring [5,6,7,8,9,10]. De-epoxi Deoxynivalenol, also known as DOM-1, Deoxynivalenol-3-glucuronide (DON-3-GlcA), and deoxynivalenol-15-glucuronide (DON-15-GlcA) are the three major DON metabolites identified in mammals. Highly sensitive analytical methods have been developed for the precise quantification of DON and its metabolites in urinary samples [11].

Vegetarians are alleged to be a high-risk group for DON exposure due to high intakes of cereals susceptible to the growth of the mycotoxin [12,13] and their proportionately higher contribution to the diet, in the absence of meat, poultry, fish, and seafood. The Vegetarian Society in the UK defines a vegetarian as “someone who lives on a diet of grains, pulses, nuts, seeds, vegetables and fruits with or without the use of dairy products and eggs. A vegetarian does not eat meat, poultry, game, fish, shellfish or by products of slaughter” [14]. The Vegan Society defines veganism as “excluding all animal flesh and animal products including milk, honey and eggs and may also exclude any products tested on animals or any clothing originating from animals” [15]. In the UK in 2012, it was estimated that 2% of adults and children were vegetarians, which equates to approximately 1.2 million people. Less than 1% reported following a vegan diet [16]. This is lower than numbers previously reported in the foods standards survey, which reported 6% of households contained at least one vegetarian member [17]. In 2005, the first French total diet study carried out an investigation to assess the exposure of the French population to mycotoxins via principal foods. The French total diet study states that vegetarians represent about 2–3% of the French population. On this basis, the ovolactovegetarian population represented 1.8%, the lactovegetarians about 1.0% and the vegans/macrobiotics about 0.2% of the French population [13].

The most common type of vegetarian diet is ovolactovegetarianism, which consists of a diet containing grains, eggs, dairy, pulses, nuts and seeds, and fruit and vegetables, as well as both dairy products and eggs. Lactovegetarians eat dairy products, but not eggs. Ovovegetarians eat eggs, but not dairy products [18].

In this study, vegetarians were classified as individuals following a vegetarian diet for over one year, excluding meat and fish but allowing animal sub-products such as butter, milk, cheese, eggs, and honey. Vegans were not excluded from the study. Pescatarians (vegetarians who also consume fish) were excluded.

The data utilised in this study is from a larger study by the European Food Safety Authority (EFSA) (*Experimental study of deoxynivalenol biomarkers in urine, GP*/*EFSA*/*CONTAM*/*2013*/*04)* [19]. In this study, DON and its metabolites were measured in urine from European population groups, including vegetarians (defined as following a vegetarian diet for more than one year), elderly, adults, adolescents, children, and pregnant women. These population groups were analysed to develop reliable information on the associations between self-reported cereal food consumption and concentrations of DON in urine. In the present study, we extracted the results to specifically analyse and compare levels of DON and its metabolites in urine samples of UK vegetarians with that of UK adults. This paper examines the DON exposure of vegetarians in a suburban setting (Hull, UK) providing data on levels of DON in human urine samples analysed by liquid chromatography-mass spectrometry (LC-MS).

## 2. Results

Thirty-two female and male vegetarians were enrolled in the study between May and October of 2014. Vegetarian females (*n* = 21) had a mean weight of 67.3 kg, height 1.64 m, and BMI 25.1 kg/m^2^. Vegetarian males (*n* = 11) had a mean weight of 73.2 kg, height 1.69 m, and BMI 25.1 kg/m^2^. Fifteen UK adult females (*n* = 15) had a mean weight of 73.1 kg, height 1.66 m, and BMI 26.7 kg/m^2^. UK adult males (*n* = 16) had a mean weight of 91.1 kg, height 1.78 m, and BMI 28.8 kg/m^2^.

Urine was positive for DON in 81% of vegetarian females on day 1 (*n* = 17) and 90.5% of females (*n* = 19) on day 2. DON was present in 77.8% (*n* = 7) of the males’ urine for both day 1 and 2. UK adult urine was positive for DON in 100% of males and females on day 1 and day 2 (*n* = 31). DOM-1 was not present on either day in any sample for UK vegetarians or adults.

For vegetarians, the mean level of DON in urine (ng/kg b.w./day) on day 1 was 855 (SD 1111) and day 2 654 (SD 654). These levels equate to up to 32% of vegetarians exceeding the recommended tolerable daily intakes (TDI) of exposure (1 µg/kg b.w./day) [4], day 1, 28% (*n* = 9) and day 2, 32% (*n* = 10).

The mean level of DON in urine (ng/kg b.w./day) for adults on day 1 was 422 (SD 291) and day 2 was 537 (SD 427), but the number of participants that exceeded the TDI was low; day 1, 3% (*n* = 1) and day 2, 9% (*n* = 3) in comparison to the vegetarian population in the UK.

Percentage coefficient variation was 69% and 80% for day 1 and 2 in adults, respectively, and 130% and 100% for vegetarians on day 1 and 2, respectively.

Non-parametric tests for correlation (Spearman’s rho) were used for the analysis in view of the skewed nature of the data. Tests for total DON (ng/mg creatinine) vs. age group, body mass index, weight, sex, and physical activity were performed. No significant correlations were found between the variables. The variable of being vegetarian did not affect urinary DON concentration.

Independent *t*-tests were performed to analyse vegetarians vs*.* UK adults. There was a significant difference between the weight of the UK adults and vegetarians, *t* = 3.15. *df* = 61, *p* = 0.005, two-tailed. Reported physical activity levels and body mass indices were not significantly different between the two groups. Due to data being skewed, the Mann-Whitney test was used to analyse for significant differences between vegetarian and UK adult biomarker results, for total DON (ng/mL urine) and DON/creatinine (ng/mg). Total DON (ng/mL urine) was significantly different on day 1 (*p* = 0.028) but not day 2 (*p* = 1.0) across the groups. DON/creatinine ng/mg was significantly different on day 1 (*p* = 0.030) but not day 2 (*p* = 0.564), alpha = 0.05, confidence interval level 95.

The mean level of total DON (ng/mL urine) concentration in UK vegetarian females on day 1 was 31.1 ng/mL (range 0–135), and on day 2 was 22.9 ng/ml (range 0–60.5). The mean total DON (ng/mL urine) concentration in UK vegetarian males on day 1 was 13.6 ng/mL (range 0–39.1), and on day 2 was 14.5 ng/mL (range 0–55.4). In UK adult females (*n* = 15), the mean total DON ng/mL on day 1 was 12.4 (range 3.3–40.6) with a median of 10.7. For day 2 (*n* = 15), the mean was 14.2 (range 0.9–36) with a median of 10.1. For UK adult males (*n* = 16), the mean on day 1 was 13.1 (range 2.2–29.4), with a median of 11.9. For day 2 (*n* = 16), the mean was 18.1 (range 5.10- 58.8), with a median of 12.6 (Table 1).

The total ng DON/mg creatinine mean for vegetarian females (*n* = 21) on day 1 was 39.9 (0–149), with a median of 22.2, and day 2 was 39.0 (0–199), with a median of 14.8. For vegetarian males (*n* = 9), the mean was 24.6 (range 0–59.3), with a median of 14.1 on day 1, and was 35.4 (range 0–157), with a median of 7.52 on day 2.

**Figure 1:**
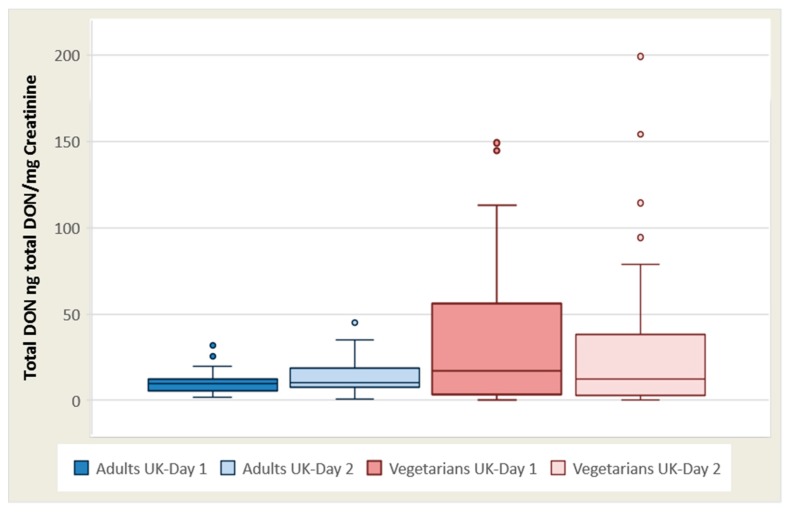
Box plot of creatinine-adjusted total DON concentrations in adults’ (blue) and vegetarians’ (red) samples for day 1 (dark) and day 2 (light). Whiskers are set from minimum to maximum values. The line inside the box is the second quartile (P50, median). Dots are suspected outliers. The bottom and the top of the box are the first and third quartiles (P25 and P75). (Brera et al., 2015).

The total DON concentration/creatinine (ng total DON/mg creatinine) mean for adult females was 12.7 (3.92–27.8), with a median of 11.3 on day 1, and on day 2 was 18.2 (1.03–66.2) with a median of 11.3. For UK adult males, the mean was 24.3 (range 0.49–153), with a median of 10.9 on day 1, and was 21.3 (range 2.48–62.3), with a median of 14.4 ng total DON/mg creatinine on day 2 (Table 1 and Figure 1).

Free DON (>LOD 0.12 ng/mL) and DON glucuronide (>LOD 0.25 ng/mL) were detected in most of the urine specimens for vegetarians, whereas for UK adults DOM-1 were not detected (LOD 0.25 ng/mL).

The mean level of free DON (ng/mg Creat) adjusted for creatinine for vegetarian females (*n* = 21) was 7.99 on day 1, and 3.59 on day 2. For vegetarian males (*n* = 11), the mean level was 5.39 on day 1, and 3.25 on day 2. The mean level of free DON (ng/mg Creat) adjusted for creatinine for adult UK females (*n* = 15) was 1.89 on day 1, and 2.77 on day 2. For adult UK males (*n* = 16), the mean level was 4.21 ng/mg creatinine on day 1, and 3.19 ng/mg Creatinine on day 2. The mean DON Glucuronide (ng/mg Creatinine) for vegetarian females was 31.88 on day 1, and 23.82 on day 2. For vegetarian males, it was 16.94 on day 1, and 12.1 on day 2. The mean DON Glucuronide (ng/mg Creatinine) for UK adult females was 10.85 ng/mg creatinine on day 1, and 15.43 ng/mg creatinine on day 2. For UK adult males, it was 20.09 ng/mg creatinine on day 1, and 18.10 ng/mg creatinine on day 2 (Table 2).

As for the consumption of food items, according to the records from Food Frequency Questionnaires (FFQ) reporting dietary habits over a 1-month period, the most common food choice for vegetarians was *Cakes and muffins* (food category *Biscuits Cakes*) with a frequency from 2–3 times per month to daily (81.2% of the group); *Wheat Bran Flakes* (food category *Breakfast cereals*) was consumed with a frequency from one time per month to daily (50.0%); *Wheat Bread and Rolls, White* (food category *Bread and breadlike*) was the most popular bread consumed with frequencies ranging from one time per month to 2–4 times per day (37.5%); *Pizza and Pizza Like Dishes* (food category *Alternative to bread*) was eaten once per month to once per week (68.7%); *Plain Dried Pasta* (food category *Pasta and pasta-like*) products were consumed food item with a frequency from one time per month to daily (59.4%). Organic muesli consumption was reported by one participant and organic bread (specifically multigrain) consumption was reported by two participants.

## 3. Discussion

The impact of mycotoxin exposure on health remains unclear, although it may contaminate up to 25% of the world’s cereal crops [20]. The high incidence of DON in the urine of UK vegetarians in 81% of vegetarian females on day 1 (*n* = 17) and 90.5% of females (*n* = 19) on day 2 as well as 77.8% (*n* = 7) of the males’ urine on day 1 confirms the significant contamination of cereal food products in the UK. In comparison to the other European sites in the original study [19], UK subjects presented with the highest concentrations of biomarkers in all subject categories apart from the elderly. Given the significant difference between the median and 75th percentilemean and range, it can be reported that very high concentrations were frequently observed (Table 1).

Surprisingly, urine was positive for DON in 100% of the urine of UK adult males and females, but only positive for DON for 77.8–90.5% of vegetarian males and females. As vegetarians are often described as a high-risk group for DON exposure, one would expect their levels to be higher if not equal to that of the UK adults.

Independent *t*-tests indicated there was a significant difference between the weight of the UK adults and vegetarians, *t* = 3.15. *df* = 61, *p* ≤ 0.005, two-tailed. Vegetarians had a lower mean BMI, but there was no statistically significant difference between the two groups.

Higher mean levels of total DON and DON/creatinine were seen in vegetarian females over both days; day 1: 31.1 ng/mL (range 0–135) and day 2: 22.9 ng/mL (range 0–60.5), and day 1: 39.9 (0–149) and day 2: 39.0 (0–199), respectively. However, when split for sex and analysed using the Mann-Whitney test, no statistical significance could be observed. When grouped for both sexes, the total DON (ng/mL urine) was significantly different for day 1 (*p* = 0.028), but not for day 2 (*p* = 1.0) across the groups. DON/creatinine ng/mg was significantly different on day 1 (*p* = 0.030) but not day 2 (*p* = 0.564), alpha = 0.05, confidence interval level 95.

In comparison to pregnant females from Hull, UK [21] who did not consume any organic foods, three vegetarian individuals in this cohort were regularly consuming organic muesli and multigrain bread. Two of the adults consumed organic products, namely wheat bran rolled flakes daily and organic pasta once a week. The socioeconomic status of individuals in the Hull region is lower than that of other areas, and this may have had some bearing on the low number of people consuming organic produce.

Previous literature on DON exposure via food consumption or the DON content of urine in vegetarians or vegans is minimal, and very few studies have been conducted in this area. A previous UK dietary survey by Turner et al*.* (2008) [22] used the UK adult National Diet and Nutrition Survey to compare 24-h urinary DON excretion with cereal intake [23]. One hundred subjects were identified for each of the following cereal consumption groups: low, medium, and high. DON was analysed in 24-h urine samples. Each participant completed a detailed 7-day weighed food diary. Of the 300 participants included in the trial, only 18 of them were vegetarian (6%). Of the 18 vegetarian individuals, four were classified as having a low cereal intake, six were classified as having a medium cereal intake, and eight were classified as having a high cereal intake. Neither ethnicity nor vegetarian status were significantly different between cereal intake groups. Despite vegetarians being regarded as at a high risk group, vegetarianism was not significantly associated with urinary DON; only sex was significantly associated with males having higher levels than females [11].

The findings of Turner et al. (2008) [11] are similar to the results of this study. When non-parametric tests were used, vegetarianism was not significantly associated with higher levels of DON in the urine. However, sex was not associated with higher levels in this study.

In 2005, the first and second French total diet studies carried out investigations to assess the exposure of the French population to mycotoxins via principal foods [13,24]. The results showed contaminant levels observed in the foods examined ‘as consumed’, rather than analysing the participants urine post food consumption. In the first French total diet study [13], 456 composite samples were prepared from 2280 individuals samples and analysed for mycotoxins; aflatoxins, ochratoxin A, patulin, trichothecenes (including DON), zearalenone, and fumonisins. The vegetarian population were considered as having a higher risk of exposure to mycotoxins in view of their greater consumption of vegetables and cereals. A 7-day consecutive food diary was recorded for 1474 adults, and portion size was calculated from the diaries by a dietitian [13]. In the vegetarian population, the average DON intake was between 320 and 410 ng/kg b.w./day (TDI equivalent of 32–41%), depending on the group studied [13].

According to our study, for UK vegetarians, the mean level of DON in urine (ng/kg b.w./day) on day 1 was 855 (SD 1111) and day 2 654 (SD 654). The proportion of subjects whose theoretical intake exceeds the TDI in France is estimated to be 0.4% in adults, 4% in children, and 4–5% in vegetarians. The levels of percentage exposure in the UK vegetarian for days 1 and 2 were 28% and 32%, respectively, fivefold that of the exposure reported in the French population [13]. Le Blanc et al*.* (2005) suggested attention should be paid to children and macrobiotics/vegans who may ingest certain mycotoxins at levels that exceed the tolerable or weekly daily intake levels [13]. The estimated average deoxynivalenol intake of the French adult population is 281 ng/kg b.w./day 1. The 95th percentile exposure was 571 ng/kg b.w./day 1 in adults (equivalent TDI of 57%). UK adult mean levels of DON in urine (ng/kg b.w./day) were comparable; day 1 = 422 (SD 291) and day 2 = 537 (SD 427). The number of UK adult participants that exceeded the TDI was low; day 1, 3% (*n* = 1) and day 2, 9% (*n* = 3) in comparison to the UK vegetarian population [4].

As there can be variability in the oral intake recorded by food diaries [25], food diaries were collected over two consecutive days to counteract this. A semi-quantitative food frequency questionnaire (FFQ) was used to determine each participant’s previous month of food intake that included an estimation of portion size and whether the food was organic or not. Food frequency questionnaires provide estimates of habitual intake [25,26]. Whilst it is acknowledged that the response to dietary recording is influenced by how complex the FFQ is, this potential error was circumvented by using two research dietitians with skills for performing the semi-structured interviews and with expertise in counseling and interviewing. The limitations of the study include the small sample size of 32 subjects and minimal organic foods being eaten by the participants. The poor intake of the organic food meant that it could not be robustly analysed as a variable and likely reflected the deprivation index of the population. This suggests that a study needs to be performed specifically designed to address this question for organic food consumption.

A major source of uncertainty for food items contributing to DON exposure could be due to portion size. The accuracy of the food data recorded could have improved by documenting actual weights consumed for correlation with DON concentration in urine. To obtain a more accurate estimation of the carryover of DON from ingested food to urine, instead of single first morning urine sample, a 24-h urine collection would be desirable.

Although the higher mean values observed, particularly amongst vegetarian women, it was found that being a vegetarian did not significantly increase levels of urinary DON; this result was in line with Turner et al. 2008 [11]. Only few studies have been conducted on DON in vegetarian and vegan populations; most data is collected as an add-on to larger general population studies. Global multi-centered large-scale studies are required to give a more varied population sample for extrapolating results.

## 4. Conclusions

This study provides distinctive data describing the levels of DON in the urine of a cohort of vegetarians and adults residing in the UK, analysed by LC-MS. This study raises concerns that a third of the vegetarian cohort maybe exceeding the recommended group tolerable daily intake. Larger scale studies with increased participant numbers and longer exposure analysis duration should take place in the future to ensure the safety of the UK vegetarian population.

## 5. Experimental Section

### 5.1. Ethics

Ethical approval was granted by the National Research Ethics Service (NRES) Committee of Yorkshire & the Humber—Leeds West (IRAS project code 147707). Microsoft excel 2013 and SPSS IBM 23 were used to analyse the data.

### 5.2. Study Population and Study Design

Recruitment target was 200 people for the UK. Of these, 30 had to be vegetarian (15%). We recruited 11 males and 21 females to achieve this target. Recruitment proved to be the most difficult for vegetarians, especially male vegetarians. The local populations were predominantly white working class; there are limited ethnic minorities in the region, such as Hindus and Buddhists who may follow a vegetarian diet for religious reasons. Vegetarian males were very difficult to find in the area. We also found through the interview process that a lot of the people who classified themselves as vegetarian were actually pescatarians i.e., a lactovegetarian that also consumes fish and fish products. They were consequently excluded from the vegetarian category.

Vegetarians were recruited by placing advertisement posters in vegetarian cafes/restaurants and via word of mouth as well as placing an approved advertisement in the local papers and an email distribution via the Hull and East Yorkshire Hospitals NHS and University of Hull portal.

The vegetarians were required to be in good health, as well as not taking any medication or on a stable dose of medications. Exclusion criteria included participating in a weight loss diet, inability to give informed consent/complete the questionnaire, eating disorders, or hospitalisation within three months of enrolment in the trial. A vegetarian was defined as an individual following the diet for more than 12 months.

### 5.3. Methods

The data in this study were extracted from a larger study [19], which collected data on the occurrence of DON and its metabolites in urine from European population groups to develop reliable information on the associations between self-reported cereal food consumption and concentrations of DON in urine. A total of 635 volunteers were recruited across Norway, Italy, and the UK. This paper focuses on the vegetarian subset of data in the UK.

A semi-structured interview to record the intake of each food category consumed over the last month, the size of portions, and whether the food was organic was performed. A validated questionnaire used in a Spanish study targeted to pregnant women [27] and the National Health and Nutrition Examination Survey (NHANES) FFQ [28] formed the basis of the FFQ used in this study. A 24-h food diary was documented by the participant over two consecutive days on the days prior to each collection of their first morning urine sample. Within each meal group, examples of food taken from each food category were tabulated with tick boxes provided to allow ease of recording as to whether the portion was small, medium, or large and whether it was organic. The urine samples were returned on the same day and centrifuged at the Hull Royal Infirmary (Hull, UK) immediately, in a refrigerated centrifuge at 2000 rpm for 10 min. The samples were stored at −80 °C.

Chemicals for analysing DON and its metabolites were as follows: ^13^C labelled DON standard (Sigma, Saint Louis, MI, USA; product number: 34128, 1.2 mL), DOM-1 (Sigma, Saint Louis, MI, USA; product number: 34135, 2 mL), DON (Sigma, Saint Louis, MI, USA; product number: D0156, 1 mg), Immunoaffinity columns DONtest WB™ (Vicam, Milford, MA, USA; product number: G1066) and β-glucuronidase (Type IX-A from *E. coli*; Sigma, Saint Louis, MI, USA; product number: G7396-2MU).

Analysis of urine samples for DON and its metabolites were carried out utilising the validated HPLC-MS/MS methodology [11]. The levels of the subjects in the UK were measured in urine samples in two steps. In brief, urine samples were removed from storage at −20 °C, allowed to thaw, and centrifuged (2000 rpm; 15 min; −4 °C). Aliquots (1 mL) were mixed with ^13^C-DON internal standard solution, to give a final concentration of 20 ng/mL.

#### 5.3.1. Laboratory Analysis

Laboratory analysis of urine samples for DON and its metabolites were carried out utilising the validated HPLC-MS/MS methodology [11]. Chemicals for analysing DON and its metabolites were as follows: ^13^C labelled DON standard (Sigma, Saint Louis, MI, USA; product number: 34128, 1.2 mL), DON (Sigma, Saint Louis, MI, USA; product number: D0156, 1 mg), β-glucuronidase (Type IX-A from *E. coli*; Sigma, Saint Louis, MI, USA; product number: G7396-2MU), DOM-1 (Sigma, Saint Louis, MI, USA; product number: 34135, 2 mL), and DONtest WB™ immunoaffinity columns (Vicam, Milford, MA, USA; product number: G1066). Equipment: Waters 2795 HPLC Separation Module (Waters Corp., Milford, MA, USA) with Quattro Micro Triple Quadrupole Mass Spectrometer (Micromass UK Ltd., Manchester, UK).

#### 5.3.2. Sample Preparation

Levels of DON and associated metabolites were measured in urine samples in two steps. Urine samples were removed from storage at −20 °C, allowed to thaw, and centrifuged (2000 rpm; −4 °C; 15 min). For each subject, two aliquots (1 mL) were prepared by mixing ^13^C-DON internal standard solution, to give a final concentration of 20 ng/mL. Aliquot 1: Total DON detection (glucuronide metabolites and free DON combined). To measure combined glucuronide metabolites of DON (deoxynivalenol-3-*O*-glucuronide and deoxynivalenol-15-*O*-glucuronide) and free DON and, each sample was adjusted to pH 6.8 and digested using β-glucuronidase solution (23,000 units, in KH2PO4 75 mM) in a shaking water bath at 37 °C for 18 h. After this period, the samples were removed, centrifuged (2000 rpm; −4 °C; 15 min), and the supernatant diluted to a final 4 mL with phosphate buffered saline (PBS, pH 7.4). The diluted urine sample was then passed through a wide bore DON immunoaffinity column. DON was eluted from columns with methanol (4 mL) and extracts were dried under vacuum using a SavantTM SpeedVacTM (Thermo Fisher Scientific Inc., Waltham, MA, USA) or equivalent and reconstituted in 10% ethanol (250 μL) for LC-MS analysis. DOM-1 was quantified on the same aliquot analysed for DON glucuronide. The data from previous experience suggests that urinary digestion using β-glucuronidase is able to release DOM-1 quantitatively. Aliquot 2: Free DON detection. The amount of free DON was extracted as before, but omitting the β-glucuronidase treatment.

#### 5.3.3. HPLC-MS Analysis—DON Determination

The separation of DON was achieved under reversed phase chromatography conditions using a Luna C_18_ column (150 × 4.6 mm, 5-μm particle size (Phenomonex, Macclesfield, UK) with a mobile phase sequence of 27 min 20% methanol, changing to a wash of 75% methanol after 10 min and reverting to 20% methanol after 16 min (flow rate 1 mL/min; injection volume 25μL). One fifth of the eluent was directed into the desolvation chamber of the MS and the remainder was pumped to waste. Selective ion recording (SIR) was used to quantify DON by reference to ^13^C-DON internal standard. The mass spectrometer conditions were as follows: capillary voltage 3.5 kV, desolvation temperature 300 °C, extraction cone voltage 3.00 V, sampling cone voltage 35.00 V, source temperature 100 °C, cone gas flow 50 L/h, collision energy 1.0, desolvation gas flow 500 L/h. Two masses both of DON ([DON-H]+, *m*/*z* 297.2 and [DON-Na]+, *m*/*z* 319.2) and ^13^C-DON ([^13^C-DON-H]+, *m*/*z* 312.2 and [^13^C-DON-Na]+, *m*/*z* 334.2) were monitored for 0.25 s each and summed to produce one total ion current peak for the internal standard and each analyte. The calibration curve was set by the injection of DON and ^13^C-DON standard solution (prepared in 10% ethanol) covering the range 2–250 ng/mL.

DON glucuronide values were calculated in addition to measuring free DON and total DON, as the difference between total DON and free DON values for each individual.

#### 5.3.4. LC-MS Analysis—DOM-1 Determination

Separation of DOM-1 was achieved using the same chromatographic column used for DON and a mobile phase sequence of 35 min 20% methanol, changing to a wash of 75% methanol after 20 min and reverting to 20% methanol after 26 min (injection volume 25 L; flow rate 1 mL/min). One fifth of the eluent was directed into the desolvation chamber of the MS and the remainder was pumped to waste. SIR was used to quantify DOM-1 by reference to the calibration curve, obtained by the injection of DOM-1 standard solutions (prepared in 10% ethanol) covering the range of 2–200 ng/mL, using least squares regression analysis. Two masses of DOM-1 ([DOM-1-H]+, *m*/*z* 281.3 and [DOM-1-Na]+, *m*/*z* 303.3) were monitored for 0.25 s each and summed to produce one total ion current peak for DOM-1.

#### 5.3.5. Creatinine Analysis

Levels of DON were adjusted for creatinine to correct for variable dilutions since only first morning samples of urine were collected. Urinary creatinine analysis was conducted using an in-house micro-titre plate assay [29]. Urine samples were diluted in water (1:20) and 100 L was loaded into a 96-well plate, in duplicate. A duplicate standard curve of creatinine concentrations was used per plate ranging from 0 μg/mL to 20 μg/mL. Then, 100 μL of alkaline picric acid solution was then added to each well, incubated at 25 °C for 30 min and read at 490 nm using a plate spectrophotometer. Both corrected and uncorrected for creatinine as total DON ng/mL urine were presented and as g total DON ng/mg creatinine in urine. The latter reporting allows a correction for different degrees of dilution of the urine between individuals.

#### 5.3.6. Dietary Analysis

The portion sizes documented into the FFQ and food diaries were converted to actual quantities (grams) prior to their entry into the FoodEx2 coding system [30,31]. There is a core list of food items or generic food descriptions that represent the minimum level of detail needed for intake or exposure assessments [32]. A harmonized database was developed to collect all the data related to the enrolled subjects concerning individual information. In total, the UK FFQ comprised of 61 cereal-based food items, each item corresponding to a FoodEx 2 code.

## Figures and Tables

**Table 1:** Total DON, free DON (%) and DON-GlcA (%) in UK urine samples in vegetarians with respect to adults.

Group Category	Day	Gender ^(b)^	Total DON, ng/mL			Total DON, ng /mg Creat ^(a)^			Free DON, (%)	DON-GlcA, (%)
			Mean	Range ^(c)^ (min–max)	P50/P75 ^(d)^	Mean	Range ^(c)^ (min–max)	P50/P75 ^(d)^	Mean	Mean
Adults (18–64 years)	1	F (15)	12.4	3.30–40.6	10.7/13.9	12.7	3.92–27.8	11.3/19.5	16	84
		M (16)	13.1	2.20–29.4	11.9/20.4	24.3	0.49–153	10.9/18.6	16	84
	2	F (15)	14.2	0.90–36.0	10.1/27.8	18.2	1.03–66.2	11.3/22.9	17	83
		M (16)	18.1	5.10–58.8	12.6/24.0	21.3	2.48–62.3	14.4/25.6	16	84
Vegetarians	1	F (21)	31.1	0–135	22.9/34.2	39.9	0–149	22.2/56.7	28	72
		M (9)	13.6	0–39.1	11.9/17.1	24.6	0–59.3	14.1/56.2	26	74
	2	F (21)	22.9	0–60.5	19.2/38.6	39.0	0–199	14.8/36.9	15	85
		M (9)	14.5	0–55.4	7.90/16.3	35.4	0–157	7.52/43.0	27	73

DON, deoxynivalenol; Creat, creatinine; DON-GlcA, deoxynivalenol glucuronide. ^(a)^ Creat, creatinine. ^(b)^ F, Female, M, Male; In parenthesis, number of subjects. ^(c)^ Min, minimum; max, maximum. ^(d)^ P50/P75, 50th/75th percentile.

**Table 2:** Mean levels of ng free DON/mg Creat, ng DON-GlcA/mg Creat, and ng DOM-1/mg Creat in urine samples over day 1 and 2 in UK in vegetarians compared to adults. Mean levels are obtained following the lower bound approach.

Group Category	Day	Gender ^(a)^	ng Free DON/mg Creat, (%)	ng DON-GlcA/mg Creat, (%)	ng DOM-1/mg Creat, (%)
Adults (18–64 years)	1	F (15)	1.89	10.85	0
		M (16)	4.21	20.09	0
	2	F (15)	2.77	15.43	0
		M (16)	3.19	18.10	0
Vegetarians	1	F (21)	7.99	31.88	0
		M (11)	5.39	16.94	0
	2	F (21)	3.59	23.82	0
		M (11)	3.25	12.1	0

DON, deoxynivalenol; DON-GlcA, deoxynivalenol glucuronide; DOM-1, de-epoxy deoxynivalenol; Creat, creatinine. ^(a)^ F, Female; M, Male; In parenthesis, number of subjects.
